# Supplementary material for: The Arabidopsis homolog of human G3BP1 is a key regulator of stomatal and apoplastic immunity
Source: Life Sci Alliance. 2018 May 31;1(2):e201800046. doi: 10.26508/lsa.201800046 (PMC6238584; doi:10.26508/lsa.201800046)
Supplement: Supplementary file 1 [file LSA-2018-00046_TableS1.pdf]

Table S1.

S. No.	ATG No.	Vector used	STOP codon	Cloned from	by	Purpose	Tag
pNR24_2	AT5G48650	pENTR-D/TOPO	without stop	PDA-12652	PCR and TOPO cloning	Gateway entry	None
pMHN275	AT5G48650	pGWB5	No stop	pNR24_2	LR Clonase	Localization/OE lines	GFP
pAA(B1)	Promoter of AT5G48650	pENTR-D/TOPO	without stop	pdg06741	PCR and TOPO cloning	Promoter Gateway entry	None
pAA(B1-2)	Promoter of AT5G48650	pGWB433	No stop	pAA(B1)	LR Clonase	Promoter analysis	Gus

Name	Sequence	Target gene	Purpose
**Cloning**			
N0030	CACCATGGATTCTACTGCTGCAACCA	AT5G48650	For cloning into Gateway pENTR-TOPO-F
N0031	CTWGTACGAGTTGATGCTGGCGA	AT5G48650	For cloning into Gateway pENTR-TOPO-R
AA013	GGGGACAAGTTTGTACAAAAAAGCAGGCTTCGAAGGAGATAGAACCtttttccggtagaccgtcgag	AT5G48650	Cloning the gene under native promoter-F
AA014	GGGGACCACTTTGTACAAGAAAGCTGGGTCTCCACCTCCGGATCMGTACGAGTTGATGCTGGC	AT5G48650	Cloning the gene under native promoter-R
AA090	CAC CTT TTT CCG GTA GAC CGT C	AT5G48650	Only promoter before ATG for cloning into Gateway pENTR-TOPO-GUS-F
AA091	TGT ATC GCC AAC TTC CAG AGA ATA AAC	AT5G48650	Only promoter before ATG for cloning into Gateway pENTR-TOPO-GUS-R

**Genotyping**			
N0107	AGATGGGGAAGTTGTTTCTGG	AT5G48650	LP1for genotyping Atg3bp1-1 for WT
N0118	CTCTACCAACTGGTGAGTCGC	AT5G48650	LP1for genotyping Atg3bp1-2 for WT
N0165	CGTTTTGCTACTTTGCTCACC	AT5G48650	RP1-for genotyping Atg3bp1-1 or HM
N0167	TGAAAGTTTATGCAGATGGGG	AT5G48650	RP1-for genotyping Atg3bp1-2 for HM
N0200	ATTTTGCCGATTTCGGAAC	AT5G48650	Primer T-DNA-LBb1.3 for SALK
N0201	TAGCATCTGAATTTCATAACCAATCTCGATACAC	AT5G48650	Primer T-DNA-LB3 for SAIL

**qPCR**			
AA155	TGGAGATTGCAACTCGGCTG	AT5G48650	AtG3BP1-F
AA156	TCTTGCGGGGCAAGGAAAAA	AT5G48650	AtG3BP1-R
AA021	ACCACCTGAAAGGAAGTACAGTG	AT3g18780	Refrence gene-Actin-F
AA022	CTGTGAACGATTCCTGGACCTGC	AT3g18780	Refrence gene-Actin-R
AA111	GGCCTTGTATAATCCCTGATGA	At4g05320	Refrence gene-UBQ10-F
AA112	AAAGAGATAACAGGAACGGAAA	At4g05320	Refrence gene-UBQ10-R
AA115	ACGGGCATAGTTCCACAAAG	AT2G19190	FRK1-F
AA116	CGTCAAAAGAACGACGATGA	AT2G19190	FRK1-R
AA117	ACAAATGGTCTGCTATAGCT	AT1G18570	MYB51-F
AA118	CTTGTGTGTAACTGGATCAA	AT1G18570	MYB51-R
AA119	GCGTAAATACGGGCAGAAAC	AT4G23550	WRKY29-F
AA120	GGTTTGGGTTGGGAAGTTTT	AT4G23550	WRKY29-R
392s	GGCGAGAAGTGAAGCTTTTG	AT5G26920	CBP60g-F
392as	GCACGGAGGATGATGTTTTT	AT5G26920	CBP60g-R
193s	AGACATCGTTGCTCTTCACG	AT1G73805	SARD1-F
193as	CACACTTACTTCTCCGGCAA	AT1G73805	SARD1-R
196s	GACGGGTATCTTTGGAGGAA	AT5G22570	WRKY38-F
196as	AAACCGGAGGATTGTCTTTG	AT5G22570	WRKY38-R
ksk-145	TATGGGGGATAAGGGGTTCT	AT1G74710	ICS1-F
ksk-146	GCCCTAGTTACAACCCGAAA	AT1G74710	ICS1-R
HA238	CTCATACACTCTGGTGGG	AT2G14610	PR1-F
HA239	TTGGCACATCCGAGTC	AT2G14610	PR1-R
ksk-155	ATCGTTGGAAATCGTGGTGT	AT3G57260	PR2-F
ksk-156	TCGGTGATCCATTCTTCACA	AT3G57260	PR2-R
R195S	CACGGAAACGTAGACGAGAA	AT1G02450	NIMIN1-F
R195AS	CCCGTACGACACTGAGAGAA	AT1G02450	NIMIN1-R
AA147	CAAGAGCCTTGATCGATCATTG	AT4G18170	WRKY28-F
AA148	GCAAGCCCAACTGTCTCATTC	AT4G18170	WRKY28-R
AA139	TTCATGGATCCAAAAATCCTAGA	AT2G46400	WRKY46-F
AA140	TGTGGTTTCCGAGATACTTCACT	AT2G46400	WRKY46-R
AA191	GGTCTTGGCGATACAAT	AT4G39030	EDS5-F
AA192	CAGCGAGTGCAGAGATC	AT4G39030	EDS5-R
N0429	ACTCCGTTAGCTTCACCGTT	AT3G59930	defensin-like protein-F
N0430	CATGCAGCATCGCTCTTCAG	AT3G59930	defensin-like protein-R
AA141	ACAGCACTTGTCTCGATTCT	AT1G21250	WAK1-F
AA142	TCTTTACGCTTGCAGCTCAT	AT1G21250	WAK1-R
AA149	ATGATCTCTTCCGTGTGCCA	AT5G46350	WRKY8-F
AA150	ATCATCAAGGCTCTTGTTTGAAGA	AT5G46350	WRKY8-R
AA143	GATCCACCGACAAGTGCTT	AT1G80840	WRKY40-F
AA144	AGGGCTGATTTGATCCCTCT	AT1G80840	WRKY40-R
AA145	CATTGGAAACCAGATGGAAG	AT4G12470	AZI1 -F
AA146	TCTGAGGGCTAACGTTCTTG	AT4G12470	AZI1 -R
AA193	TTAGCCGTTGAAGCTCT	AT3G52430	PAD4 -F
AA194	ATGCATCGCAACGATCT	AT3G52430	PAD4 -R
AA195	CTGAGTTAGCCGGTGT	AT3G48090	EDS1-F
AA196	TTTCATGTACGGCCCTG	AT3G48090	EDS1-R
AA183	GCATCTCGTCGCTCAG	At1g52890	ANAC019-F
AA184	CTCGACTTCCTCCTCCG	At1g52890	ANAC019-R
AA185	GCGCTGCCTCATAGTC	At3g15500	ANAC055-F
AA186	CGAGGAATCCCCTCAGT	At3g15500	ANAC055-R
AA187	TGGGTGTTGTGTCGAAT	At4G27410	ANAC072-F
AA188	ATCGTAACCACCGTAACT	At4G27410	ANAC072-R
