# Supplementary material for: The Arabidopsis homolog of human G3BP1 is a key regulator of stomatal and apoplastic immunity
Source: Life Sci Alliance. 2018 May 31;1(2):e201800046. doi: 10.26508/lsa.201800046 (PMC6238584; doi:10.26508/lsa.201800046)
Supplement: Supplementary file 2 [file LSA-2018-00046_Preparation_of_compounds.pdf]

Supplementary Information

Materials and methods

AtG3BP1 cloning and generation of *AtG3BP1* overexpression transgenic plants

The coding sequence (CDS) of *AtG3BP1* was amplified from a cDNA clone (pda12652 - RIKEN Biosciences) using primers spanning the ATG to the stop codon by PCR with Phusion High-Fidelity DNA Polymerase (New England Biolabs). Amplicon was purified and cloned into Gateway entry vector pENTR-D/Topo (Invitrogen) to generate pNR 24-2 (without stop codon) and subsequently cloned into the Gateway-compatible binary expression destination vector pGWB5 containing GFP tag driven by cauliflower mosaic virus 35S promoter by LR reaction following the manufacturer's instructions (Invitrogen) to obtain pMHN 275.

Transgenic *Arabidopsis* overexpressor stable lines were generated by floral dip transformation using *Agrobacterium tumefaciens* C58 harboring pMHN 275 (Zhang, Henriques et al., 2006). Transgenic plants were selected on ½ MS medium containing 50 µM hygromycin and propagated to obtain homozygotic T3 lines. All primer sequences are shown in Supplementary Table 1.

MAMP and hormone treatments

Flg22 peptide (QRLSTGSRINSAKDDAAGLQIA) was synthesized (GenScript Inc.,) and used at the concentration 1 µM for the treatment. ABA was used at the concentration of 20 µM and SA was used at the concentration of 100 µM. Water or Ethanol only treatment was used as mock treatment.

Gus staining and subcellular localization assays

To construct *ProAtG3BP1*::*Gus,* the native promoter of *AtG3BP1* (1 kb upstream of the start codon) was PCR amplified from a RIKEN TAC clone (pdg06741 – RIKEN Biosciences) and subcloned to pENTR-D/Topo (Invitrogen) to generate pAA(B1) (without stop codon) and subsequently recombined into pGWB433 vector by LR reaction following the manufacturer's instructions to obtain pAA(B1-2). The construct *ProAtG3BP1*::*Gus* was confirmed by sequencing and used to stably transform Col-0

via floral dip-inoculation using *Agrobacterium tumefaciens* C58. Transgenic plants were selected on ½ MS medium containing 50 µM kanamycin. Glucuronidase activity was determined on seedlings of 8 – 14 days and staining was carried out from 1 – 12 hours as described in (Zimmerli, Stein et al., 2004). Pictures of whole seedlings stained with GUS were taken using Nikon SMZ25 stereomicroscope. Magnified images of shoot and root stained with GUS were taken with Zeiss AX10 Imager Z2. All primer sequences are shown in Supplementary Table 1.

Subcellular localization assay was performed using 8-day-old roots and 12 days old leaves of stable transgenic T3 homozygous lines of *Arabidopsis* plants overexpressing 35S::AtG3BP1-GFP. Samples were visualized for GFP expression using a confocal laser-scanning microscope imaging system (Zeiss).

Transient expression in N. benthamiana leaf epidermal cells

GFP-tagged AtG3BP1 and CFP-tagged Serrate were transformed into Agrobacterium tumefaciens C58C1 strain by electroporation. Agrobacterium cultures from glycerol stocks were inoculated in 10 ml of LB medium with appropriate antibiotics and incubated for 24 h at 28°C with agitation. Each culture was pelleted and resuspended in infiltration buffer (10mM MgCl2, 10 mM MES pH 5.7, 150 µM acetosyringone) to an OD600 of 1.5 and kept in the dark for 3h. The P19 viral suppressor of gene silencing was also co-expressed to prevent silencing of transiently expressed proteins (Voinnet, Rivas et al., 2003). 500 µL of each bacterial culture were mixed and co-infiltrated into 4-week old N. benthamiana leaves. GFP and CFP fluorescence was visualized after 3 days using a laser scanning microscope imaging system (Zeiss).

Immunoblot

Total protein from wild type and 35S::AtG3BP1-GFP (OE3) 14 days old seedlings were extracted from (80-100 mg) plant material and frozen in liquid nitrogen. Samples were homogenized to a fine powered using a Tissue-Lyser. Frozen plant powder was resuspended in extraction buffer containing 50 mM Tris-HCL pH7.5, 150 mM Nacl, 0.1% NP40, 5 mM EGTA, 0.1 mM DTT, vortexed and incubated on ice for 15 min. Extract was then centrifuged at high speed for 15 min at 4 °C. The

supernatant (150 µL) was collected and protein quantification was carried out by Bradford assay (5 µL of the supernatant) (B6916, Sigma-Aldrich). Finally, protein amount was normalized for all samples and denatured with Laemmli 6X buffer by boiling at 75° C for 10 min. Samples were resolved by SDS-PAGE using Mini-Protean system (BIO-RAD) at a constant amperage 15 mA and transferred onto ethanol-activated PVDF membranes (GE Healthcare) for 120 min at a constant voltage of 100 V. Blots were blocked with 5 % milk in 1 x TBST at RT for 1 h. Then, blots were probed with anti-GFP antibody O/N at 4 °C on a rotary shaker. The membranes were washed three times for (5 min each) with 1 x TBST and then were incubated with the appropriate secondary antibody conjugated to horseradish peroxidase with 2 % milk in 1 x TBST for 1 h. The membranes were washed again three times for (5 min each) with 1 x TBST and the antigen-antibody interactions were detected using with enhance chemiluminescence reagent (ECL Prime, GE Healthcare) using a ChemoDoc MP Imaging System (BIO-RAD. Ponceau-S staining of blots was then carried out for protein visualization

Bacterial pathogen infection assays

The bacterial strains used in this study were *Pseudomonas syringae* pv.*tomato* DC3000, *Pst* DC3000 *hrcC*-, and *Pst* DC3000 *COR*-. Syringe and spray inoculations were performed as described (Katagiri, Thilmony et al., 2002). Briefly, five-week-old *Arabidopsis* WT and mutant plants were grown on jiffy 7 under short day conditions (8 h/16 h) at 23°C and 60% RH and challenged with bacterial strains (OD600nm = 0.2 in 10 mM MgCl2 containing 0.04% (v/v) Silwet L-77) by either spray inoculation or infiltration into leaves with a needleless syringe. Disease symptoms were evaluated at 3 and 48 or 72 h post infection (hpi). For bacterial titers, leaf discs (0.5 cm diameter) from three different leaves per plant (five plants per genotype for 3 hpi and 10 plants for 48 or 72 hpi were used as technical replicates per experiment) were harvested, washed, and then bacteria were extracted using 10 mM $MgCl_2$ containing 0.04% (v/v) Silwet L-77. Quantification was done by plating appropriate dilutions on LB agar media containing rifampicin (50 mg/l) and incubated at 28°C for 2 days, after which the bacterial numbers were counted.

Stomatal aperture assay

Abaxial epidermal peels from fully expanded leaves of 5-week-old *Arabidopsis* WT

and mutant plants were floated on stomatal opening buffer solution (10 mM KCl in 10 mM MES buffer, pH 6.15 (KOH)). The peels were incubated for 2 h under light (100 μmol $m^{-2}s^{-1}$) to open stomata before stomatal aperture measurement was performed as described (Desclos-Theveniau, Arnaud et al., 2012). The treatments with the MAMP flg22 and with the hormones ABA or SA were then performed at the indicated concentrations. Water/ethanol was added as mock control. For *Pst* DC3000 and *Pst* DC3000 *COR*- inoculation, bacterial suspensions at an OD600 of 0.2 with 0.04% (v/v) Silwet L-77 were added to the stomata opening buffer. The peels were mounted on slides and microscopic images of stomata were taken at the indicated time points using a Zeiss AX10 Imager Z2. Internal width of stomatal apertures and mean pore depth, which is assumed to be equivalent to guard cell width, were measured as described in (Dow & Bergmann, 2014) using the "measure" function of ImageJ (http://rsb.info.nih.gov/ij/). Peels from at least 3 plants were used for each sample and forty images were used per biological replicate.

Oxidative burst assay

Rapid and transient production of ROS was monitored by a luminol-based assay (Huang, Desclos-Theveniau et al., 2013). Twelve leaf discs (0.4 cm diameter) from 4-week-old *Arabidopsis* WT and mutant plants were incubated adaxial side up in 150 μL of sterile water in a white 96-well plate (Thermo Fisher) overnight. Water was then replaced with 100 μL of reaction solution containing 50 μM of luminol, 10 μg/mL of horseradish peroxidase (Sigma), and supplemented either with 1 μM of flg22 or water as a mock control. ROS evaluation was conducted immediately at 1 min interval reading time after the addition of MAMPs for a period of 40 min using TECAN Infinite 200 PRO microplate reader; signal integration time was 0.5 s. ROS measurements were expressed as means of RLU (Relative Light Units).

Superoxide radical and hydrogen peroxide staining

Fully expanded leaves of 5-week-old *Arabidopsis* WT and mutant plants were stained with either nitroblue tetrazolium (NBT) (N6876, Sigma-Aldrich) or 3,3'diaminobenzidine (DAB) (D5637, Sigma-Aldrich) for superoxide radical and hydrogen peroxide staining method, respectively, as described in (Ramel, Sulmon et

al., 2009). Leaves were then mounted on slides with 50% glycerol. Pictures were taken using Nikon SMZ25 stereomicroscope.

RNA extraction and gene expression analysis

For MAMP-induced defense gene expression, 14-day-old Arabidopsis WT and *Atg3bp1-1* mutant seedlings grown on ½ MS agar were transferred on liquid ½ MS overnight before flg22 treatment. Seedlings were treated with either 1 µM flg22 or water (mock) for 1 hour. For RNA-seq analysis, 14-day-old Arabidopsis WT and mutant seedlings were spray inoculated with *Pseudomonas syringae* pv *tomato* DC3000-*hrcC*- for 24 h. Total RNA was extracted using NucleoSpin® Plant RNA kit and first-strand complementary DNA was synthesized from 1 µg of total RNA using reverse transcriptase, SuperScript® III First-Strand Synthesis SuperMix kit (Invitrogen). Quantitative RT-PCR (qRT-PCR) analysis was performed using SsoAdvanced Universal SYBR Green Supermix (Bio-Rad). Data were analyzed using Bio-Rad CFX manager software. AT3g18780 (*Actin*) and At4g05320 (*UBQ10*) were used as reference genes for normalization of gene expression levels in all samples. Normalized gene expression was expressed relative to wild-type controls in each experiment (expression level = 1). Primers are listed in Supplementary Table 1.

RNA-seq and Data Analysis

Three independent biological replicates were carried out for RNA-seq analysis. RNA-seq library preparation and sequencing was performed on HiSeq 4000 platform resulting in 151 bps paired-end reads. Approximately 40 million reads were obtained for each sample. Reads were quality checked using FASTQC v0.11.5 (Bioinformatics, November 5, 2017). Adapters and reads with low sequencing quality were filtered using Trimmomatic 0.36 (Bolger, Lohse et al., 2014), retaining first 100 bps and by using other default settings for paired-end sequences. The trimmed reads were then aligned to the Arabidopsis reference genome (TAIR10) using Tophat v2.1.1 (Trapnell, Pachter et al., 2009, Trapnell, Roberts et al., 2012) with –N 2 –g 1. The annotation file was provided as reference for reads alignment. MultibamSummary from deepTools2 package (Ramirez, Ryan et al., 2016) was used on the bam files derived from the previous step, to check for the correlation between the replicates. Summary of read counts at gene level was calculated using featureCounts v1.5.1 (Liao, Smyth et al., 2014). Cufflinks v.2.2.1 (Trapnell et al.,

2012) was used to calculate the FPKM values for individual replicates and CuffDiff v2.2.1 (Trapnell et al., 2012) with quartile normalization to find the significant differential gene expression. Genes with 2-fold change and P value <= 0.05 were considered as significantly different between samples with and without *Pst hrcC-* treatment. Hierarchical clustering of these genes was performed using Mev v4.8.1 (Howe, Sinha et al., 2011). GO term enrichment in each gene list was carried out using AgriGO (Du, Zhou et al., 2010) with a cutoff for significant enrichment is P value < 0.01 and calculation false discovery rate < 0.5. Venny diagrams were generated using (http://bioinfogp.cnb.csic.es/tools/venny/).

Salicylic acid quantification

Arabidopsis WT and *Atg3bp1-1* mutant 14-day-old seedlings were harvested into liquid nitrogen, freeze dried, and ground to a fine powder. Total SA was extracted and quantified by LC-MS/MS based on a comparison with the standard SA as previously described (Forcat, Bennett et al., 2008)

Statistical Analysis

Microsoft Excel software was used for statistical analysis. Statistically significant groups were determined by a Mann Whitney test sterisks indicate significant differences from the WT as * for P ≤ 0.05, ** for P ≤ 0.01, and *** for P ≤ 0.001.

References

Bioinformatics B (November 5, 2017) FastQC A Quality Control tool for High Throughput Sequence Data. (n.d.). . In

Bolger AM, Lohse M, Usadel B (2014) Trimmomatic: a flexible trimmer for Illumina sequence data. Bioinformatics (Oxford, England) 30: 2114-20

Desclos-Theveniau M, Arnaud D, Huang TY, Lin GJ, Chen WY, Lin YC, Zimmerli L (2012) The Arabidopsis lectin receptor kinase LecRK-V.5 represses stomatal immunity induced by Pseudomonas syringae pv. tomato DC3000. PLoS pathogens 8: e1002513

Dow GJ, Bergmann DC (2014) Patterning and processes: how stomatal development defines physiological potential. Current opinion in plant biology 21: 67-74

Du Z, Zhou X, Ling Y, Zhang Z, Su Z (2010) agriGO: a GO analysis toolkit for the agricultural community. Nucleic acids research 38: W64-70

Forcat S, Bennett MH, Mansfield JW, Grant MR (2008) A rapid and robust method for simultaneously measuring changes in the phytohormones ABA, JA and SA in plants following biotic and abiotic stress. Plant methods 4: 16

Howe EA, Sinha R, Schlauch D, Quackenbush J (2011) RNA-Seq analysis in MeV. Bioinformatics (Oxford, England) 27: 3209-10

Huang TY, Desclos-Theveniau M, Chien CT, Zimmerli L (2013) Arabidopsis thaliana transgenics overexpressing IBR3 show enhanced susceptibility to the bacterium Pseudomonas syringae. Plant biology (Stuttgart, Germany) 15: 832-40

Katagiri F, Thilmony R, He SY (2002) The Arabidopsis thaliana-pseudomonas syringae interaction. The Arabidopsis book 1: e0039

Liao Y, Smyth GK, Shi W (2014) featureCounts: an efficient general purpose program for assigning sequence reads to genomic features. Bioinformatics (Oxford, England) 30: 923-30

Ramel F, Sulmon C, Bogard M, Couee I, Gouesbet G (2009) Differential patterns of reactive oxygen species and antioxidative mechanisms during atrazine injury and sucrose-induced tolerance in Arabidopsis thaliana plantlets. BMC plant biology 9: 28

Ramirez F, Ryan DP, Gruning B, Bhardwaj V, Kilpert F, Richter AS, Heyne S, Dundar F, Manke T (2016) deepTools2: a next generation web server for deep-sequencing data analysis. Nucleic acids research 44: W160-5

Trapnell C, Pachter L, Salzberg SL (2009) TopHat: discovering splice junctions with RNA-Seq. Bioinformatics (Oxford, England) 25: 1105-11

Trapnell C, Roberts A, Goff L, Pertea G, Kim D, Kelley DR, Pimentel H, Salzberg SL, Rinn JL, Pachter L (2012) Differential gene and transcript expression analysis of RNA-seq experiments with TopHat and Cufflinks. Nature protocols 7: 562-78

Voinnet O, Rivas S, Mestre P, Baulcombe D (2003) An enhanced transient expression system in plants based on suppression of gene silencing by the p19 protein of tomato bushy stunt virus. The Plant journal : for cell and molecular biology 33: 949-56

Zhang X, Henriques R, Lin SS, Niu QW, Chua NH (2006) Agrobacterium-mediated transformation of Arabidopsis thaliana using the floral dip method. Nature protocols 1: 641-6

Zimmerli L, Stein M, Lipka V, Schulze-Lefert P, Somerville S (2004) Host and non-host pathogens elicit different jasmonate/ethylene responses in Arabidopsis. The Plant journal : for cell and molecular biology 40: 633-46
